# Black and White older adults’ end-of-life experiences: does hospice use mitigate racial disparities?

**DOI:** 10.1093/geronb/gbaf137

**Published:** 2025-07-26

**Authors:** Clifford Ross, Brina Ratangee, Emily Schuler, Zheng Lian, Benmun Damul, Deborah Carr, Lucie Kalousová

**Affiliations:** Departments of Medicine, Health, and Society and Sociology, Vanderbilt University, Nashville, Tennessee, United States; Department of Medicine, Health, and Society, Vanderbilt University, Nashville, Tennessee, United States; Department of Medicine, Health, and Society, Vanderbilt University, Nashville, Tennessee, United States; Department of Sociology, Vanderbilt University, Nashville, Tennessee, United States; Institute for Medicine and Public Health, Vanderbilt University School of Medicine, Nashville, Tennessee, United States; Department of Sociology and Center for Innovation in Social Science, Boston University, Boston, Massachusetts, United States; Departments of Medicine, Health, and Society and Sociology, Vanderbilt University, Nashville, Tennessee, United States

**Keywords:** Death quality, Racial disparities, Hospice care, Proxy-reported outcomes

## Abstract

**Objectives:**

Racial disparities in end-of-life care are well documented, but less is known about how these inequalities shape assessments of death quality. This study examines Black–White differences in two core dimensions of proxy-reported end-of-life experience: perceived death quality and perceived care concordance. We also assess whether hospice care moderates racial differences in death quality outcomes.

**Methods:**

Data are from the Health and Retirement Study Core and Exit Interviews conducted between January 2018 and September 2023. Our analytic sample included 2,498 decedents (450 Black, 2,048 White). Multivariable ordinary least squares and logistic regression models are used to estimate the associations between race, hospice use, and our two end-of-life experience outcomes.

**Results:**

Proxies for Black decedents reported higher perceived death quality than those for White decedents, despite evidence of greater structural disadvantage. However, perceived care concordance was significantly lower among Black decedents. Hospice care was associated with improved perceived death quality for Black decedents but not for Whites. When accounting for socioeconomic and death experience controls, hospice care did not moderate perceived care concordance.

**Discussion:**

Our findings highlight the importance of considering expectations, context, and reference group comparisons when interpreting subjective end-of-life measures. Expanding equitable access to high-quality hospice care may help reduce persistent racial disparities, but interventions must also address how care is experienced, evaluated, and aligned with individual preferences.

Racial disparities in health care quality and access in the United States are well established and are important contributors to Black–White disparities in morbidity and mortality (Phelan & Link, 2015). Black adults have a higher risk of most chronic diseases, an earlier age of onset of those diseases, and premature mortality relative to their White counterparts. These disparities stem from a range of structural factors—including lower rates of health insurance among Black adults, fewer economic resources to pay for preventive care, limited access to high-­quality providers in racially segregated neighborhoods, and experiences of discrimination and compromised care quality in clinical settings—and persist even after controlling for multiple indicators of socioeconomic status (Colen et al., 2019; Williams et al., 2019). While health disparities and racial health inequality have been well documented, less is known about how these inequalities shape the dying experience, and how contextual factors may exacerbate or mitigate racial disparities in the quality of end-of-life care. One such contextual factor is the use of hospice care, which emphasizes comfort rather than intrusive treatments at the end-of-life. Extensive research documents the beneficial effects of hospice for quality of end-of-life care (Kleinpell et al., 2019; Anhang Price et al., 2025); however, Black persons use hospice at much lower rates than their White counterparts, reflecting both attitudinal and structural obstacles (Rosenfeld et al., 2007; Tate et al., 2025).

To understand end-of-life health outcomes, we must also consider the challenges of interpreting subjective health measures. Past research has shown that Black Americans tend to overestimate their health when reporting certain health measures, like self-rated health (Bernstein & Sasson, 2023; Lo et al., 2013; Mirowsky, 1999). The paradox of projecting better physical health despite generally poorer health outcomes and earlier mortality is often attributed to individuals measuring their health in comparison to the health of their peers or those in their immediate environment, leading to older adults reporting themselves as younger and healthier than they generally are (Sayag & Kavé, 2022). Similarly, when measures of death quality are reported, primarily by proxies, they may be done so in the context of deaths that the individual has seen. In contexts where structural disadvantages persist and access to care is limited, deaths appear less painful, less traumatic, or where an individual maintains health care agency may be rated positively, even if it may fall short of established clinical standards for high-quality end-of-life care. Understanding these dynamics is important for interpreting racial patterns in the quality of death, and it is therefore important to assess potential Black–White differences using multiple measures of death quality. Doing so will give more accurate assessments of the ways that health disparities may persist throughout the life course and into end-of-life care.

Addressing this, our study uses national representative data from the Health and Retirement Study (HRS) to examine Black–White differences in two core dimensions of end-of-life care as reported by recent decedents’ proxies: the quality of care received, and the extent to which one’s treatment preferences are heeded. We further evaluate whether these associations are moderated by hospice use. Understanding racial disparities in core dimensions of death quality is essential for identifying structural inequalities in end-of-life care and for informing more equitable health and policy interventions.

## Background

### Defining death quality

Advances in medical technologies and changes in the leading causes of death over the past century have transformed death and dying from a largely sudden, unexpected event following acute illness to a more protracted transition often following a period of chronic illness, especially in old age (Carr, 2012; Carr & Luth, 2019). This transition has led to an increased interest in understanding and improving the quality of death. A good death is generally agreed to be “free from avoidable distress and suffering for patients, families, and caregivers; in alignment with patients’ and families’ wishes; and reasonably consistent with clinical, cultural, and ethical standards” (Emanuel & Emanuel, 1998).

Specific dimensions of a good death include few or well-­managed physical, psychological, and cognitive symptoms; having one’s psychological, spiritual, existential, and caregiving needs met; not posing a psychological or financial burden on family members; patient and family autonomy in the decision-making process; and having one’s hopes and preferences realized. Importantly, these definitions provide implicit guidance regarding potential sites for intervention, such as the use of hospice or palliative care to address symptoms of discomfort or promoting the use of advance care planning to ensure that patients and their families can articulate their preferences for care while they are able to do so (Institute of Medicine, 2015).

High-quality end-of-life care is essential for the well-being of dying patients as well as the family members and caregivers engaged in that care (e.g., Carr & Luth, 2019). A small yet growing body of literature has documented racial differences in end-of-life care, including end-of-life care access, place of death, and pain management (Johnson, 2013). Black Americans have been found to receive more aggressive medical care at the end of life, have lower rates of hospice use, receive less adequate pain management, receive less counseling about end-of-life treatment options, and be less likely to engage in advance care planning, which may significantly impact their overall death quality (Bazargan & Bazargan-Hejazi, 2021; Johnson, 2013).

While factors such as pain management, clear decision making, coordination of care, and preparation for death are commonly used to assess overall death quality across all racial and ethnic groups, research suggests that members of racial and ethnic minority groups may differ in how they prioritize them. Compared to White Americans, Black Americans are more likely to oppose non-life-sustaining palliative drugs and prefer to receive intensive medical care, including enteral nutrition, transfusions, mechanical ventilation, and ICU care (Boyce-­Fappiano et al., 2021; Braun et al., 2008). Some sociologists have further argued that the conceptualization of a good death disproportionately reflects the values of White middle- and upper-class cultures, as seen through the theme of death acceptance aligned with what Western medical professionals’ notions of a good death (Conway, 2012; Hauschildt, 2024; Howarth, 2007). Despite growing recognition of racial disparities in end-of-life care, there is limited understanding of how these inequities manifest in subjective assessments of death. Most existing research emphasizes structural indicators, such as place of death, treatment received, or decision making, but pays less attention to how deaths are experienced and evaluated. This gap matters, as subjective measures may reflect different dimensions of care quality, including relational and cultural expectations. While Black Americans are more likely to experience end-of-life health care treatment associated with poorer death quality (O’Mahony et al., 2021), how this translates to subjective measures of reported death quality has yet to be explored.

### Measuring death quality

There are multiple ways that death quality is measured in the current literature. These usually include clinical measures of the dying experience and/or proxy reports, usually from a spouse or close family member. Clinical measures usually include formalized health services like pain management, quality of end-of-life care, and dying in one’s home rather than in the hospital (Carr & Luth, 2019).

Proxy assessments of death quality are generally collected by asking respondent proxies to rate the quality of the decedents’ death experiences after the death has occurred. While both have strengths and weaknesses, each presents unique methodological challenges. When measuring death quality from proxy-reported measures, cultural differences may influence perceptions of a good death. Some proxies may place less importance on prioritizing pain management or death location and may instead prioritize other factors like spiritual fulfillment or social togetherness. Additionally, depending on the relationship of the proxy, recall bias and emotional distress can affect the accuracy of their reports. Therefore, it is important to consider the perspectives of the reporters when looking at proxy-­reported death quality measures.

### Current study

The current study contributes to the literature on racial disparities in death quality by incorporating two distinct measures of death quality—perceived death quality and perceived care concordance—and examining associations between hospice care and death quality. Using high-quality survey data from exit interviews collected by the HRS between January 2018 and September 2023, we investigated the following research questions: (a) To what extent do perceived death quality and perceived care concordance differ between Black and White Americans? (b) Does receipt of hospice care reduce Black–White disparities in perceived death quality and perceived care concordance?

We hypothesize that proxies for Black decedents will report lower quality deaths on both measures and that hospice care services will moderate this relationship. The findings of this study contribute significantly to the health disparities and death quality literature by furthering our understanding about how racial groups experience and perceive death quality.

## Method

### Data

We used data from the HRS, a longitudinal survey of U.S. adults ages 51 years and older, as well as their spouses of any age (Sonnega et al., 2014). Our analyses used both the longitudinal core study data provided by HRS participants (Bugliari et al., 2023), and exit interview data provided by proxies of the respondents following their deaths (Health and Retirement Study, 2023). Approximately 1 year after an HRS participant has died, an interview with a respondent proxy (previously appointed by the participant) collects information about the respondent’s last year of life, as well as their death experiences. These data are ideally suited for studying disparities in the perceived quality of end-of-life experiences (Health and Retirement Study, 2020, 2022).

We used exit survey data from the 2018, 2020, and 2022 waves. In 2018, exit interviews began to ask proxies to rate the quality of the respondent’s last week of life. Because they directly ask about death quality, these data enable us to document the quality of end-of-life experiences., whether observed experiences were similar for Black and White decedents, and if death quality may be moderated by hospice use.

### Measures

#### Dependent variables

We focus on two proxy-reported dimensions of end-of-life experiences: (a) the perceived quality of the decedent’s last week of life (“perceived death quality”), and (b) the extent to which care was reported to be concordant with the decedent’s wishes (“perceived care concordance”). To measure the first dimension, proxy respondents were asked, “On a scale of zero to 10, where zero is the worst possible and 10 is the best possible, in your opinion, how would you rate the overall quality of [deceased R’s] last week of life?”

For the second, proxies were asked, “Thinking about [his/her] experiences with the health care system in the last year before [he/she] died, how often were [his/her] wishes for care taken into account, never, sometimes, usually, or always?” Based on the skewed distribution of responses, where 5% reported never having their wishes taken into account, 13% reported sometimes, 27% reported usually, and 55% reported always, we constructed a dichotomous measure of never/sometimes versus usually/always.

#### Main predictors

Our goal is to examine racial disparities in the quality of death and the extent to which the use of hospice might mitigate or amplify these disparities. Thus, our focal predictor variables are race and receipt of hospice care. Race refers to whether a respondent self-identified as Black or White (reference group) in the HRS core data collected when they were alive. Our decedent sample is primarily White (82%).

Hospice care was assessed in two steps. First, proxies were asked: “In the last two years, had [he/she] received any hospice services?” Second: “How long (in total) were hospice services in place before [his/her] death?” If a proxy reported that the decedent received hospice care and that care was three or more days, they were categorized as receiving hospice care. We selected this cutoff based on research showing that very short hospice stays of three days or less do not adequately meet the needs of dying patients or their family members (e.g., Kris et al., 2006). Proxies for White decedents report receipt of hospice care more often, with 42% of White decedents reported as having received hospice care for at least three days, compared to only 25% of Black decedents.

#### Control variables

We adjusted for demographic, socioeconomic, and death circumstances characteristics that may confound observed statistical associations among race, hospice care, and quality of death. Covariates were obtained from proxies during the exit interview and from decedents in the core interview from the final HRS wave in which they participated.

Demographic and socioeconomic covariates include gender, Hispanic ethnicity, marital status (married/partnered; never married; divorced/separated; widowed), educational attainment (less than high school; high school or equivalent; some college; 4-year degree or more), and total net worth at the time of death (in U.S. dollars; negative or zero; $1–49.9k; $50k–99.9k; $100k–249.9k; $250k–499.9k; and $500k+), and whether or not the respondent had a living will. Additionally, we control for factors that may impact the death experience, including whether the decedent died in the hospital and if they received palliative care before death. While palliative care and hospice care can often have similar goals, those receiving palliative care generally are also experiencing a lot of general discomfort (National Institute on Aging, 2021). In our sample, around 30% of those receiving hospice care also received palliative care at one point.

Additionally, due to the novel effects on health and health care, we include a measure of whether the decedent died before versus during the COVID-19 pandemic. If the proxy reported the date of death as during or after March 2020, the respondent was coded as having died during the COVID-19 pandemic. Although the timing and intensity of COVID’s impact varied by region, the CDC COVID-19 Response Team (2020) confirmed that “by mid-March [2020], all 50 states, the District of Columbia (DC) and four U.S. territories had reported cases of COVID-19.” In our sample, there were no racial differences in death timing, with 60% of deaths, both Black and White, occurring before March 2020.

Death circumstances variables were taken from exit survey data, these include age at death (in years), one or more living children (yes; no), relationship between the respondent and the proxy (spouse; child/child-in-law; other relative; other or professional), whether the death was expected at the time it occurred (yes; no), and duration between the death and exit interview.

### Analytic sample

Proxy reports of end-of-life experiences are available for 3,083 HRS respondents who died between 2014 and 2023. Item-­specific missing data for three variables (living will, marital status, and total assets) were imputed with self-reports from the decedent at the most recent of the core surveys in which they participated. For example, if the proxy was unsure if the decedent had a living will, we imputed that information from the last time the decedent was interviewed and answered the living will question.

Respondents were dropped from the analysis if they died in 2020 and their proxies did not report a month of death (*n* = 6). We limited our analytic sample to Black and White respondents because our focus is on Black–White disparities, and we therefore dropped respondents identified as Asian, Native American, or other (*n* = 163). We also excluded respondents who were missing on one or both death quality measures (*n* = 268) and those missing hospice care enrollment information (*n* = 64). Finally, we dropped respondents who were missing on one or more of our control variables that could not be imputed from core data (*n* = 84). Our final analytic sample size is (*N* = 2,498; 450 Black and 2,048 White decedents).

### Analytic plan

We first present descriptive statistics for all measures used in the analysis and conduct *t*-tests to evaluate Black–White differences. We then estimate multivariable ordinary least squares (OLS) and binary logistic regression models to document the independent associations of race and hospice care on the two end-of-life dimensions: perceived quality of death and the receipt of care concordant with patient wishes, respectively. We test the association between hospice care and quality of death using moderation analysis by evaluating the interaction between hospice care and race. All analyses were performed using Stata 18 (StataCorp, 2023).

## Results

### Bivariate analyses

Descriptive statistics, for the full sample and stratified by race, are shown in Table 1. Proxy respondents perceived decedents’ quality of death to be of moderate quality (Mean = 5.3 on 10-point scale), with deaths of Black patients rated as significantly better than deaths of White patients (6.1 vs 5.1, *p <* .001). However, Black decedents were significantly less likely to have their health care wishes always/usually met (72% vs 84%, *p <* .001) and were significantly more likely to die unexpectedly (57% vs 40%, *p <* .001) and to die in a hospital (39% vs 26%, *p <* .001). They were also significantly more likely to die without a living will (79% vs 44%, *p <* .001). Black decedents also died significantly younger than Whites (77.0 vs 83.9, *p <* .001), consistent with the vast literature documenting racial disparities in life expectancy. We also detect socioeconomic disparities, consistent with previous research documenting Black older adults’ lower levels of educational attainment and wealth relative to their White counterparts. Black decedents were also significantly less likely than Whites to be married at the time of death. We detected slight Black–White differences in who served as their proxy, with White decedents more likely to have a child and Black decedents more likely to have a relative other than a spouse or child to complete the proxy interview.

**Table 1.** Descriptive statistics by race, health, and retirement study (*N* = 2,498).

Variable	Full sample (*N* = 2,498)	Black (*n* = 450)	White (*n* = 2,048)	*p*
**Outcome variables**	100%	18.0%	82.0%	
Proxy-rated death quality score (range: 0 (worst) to 10 (best)	5.3 (3.5)	6.1 (3.4)	5.1 (3.5)	<.001
End of life wishes never/sometimes met (ref = Always/Usually)	17.9%	27.1%	15.8%	<.001
**Main predictor**				
Received hospice care (ref = No)	38.6%	24.9%	41.6%	<.001
**Control variables**				
Women (ref = Men)	56.5%	58.2%	56.1%	<.001
Is Hispanic (ref = No)	6.9%	2.4%	7.9%	<.001
Age at death (years)	82.6 (10.2)	77.0 (11.1)	83.9 (9.6)	<.001
COVID-19 Death (ref = Death before 2020)	39.2%	40.2%	39.0%	.658
No living will (ref = Had living will)	50.4%	78.7%	44.2%	<.001
One or more living? Child(ren) (ref = No)	94.0%	93.0%	94.2%	.270
Marital status				<.001
Married/partnered	40.2%	28.0%	42.9%	
Never married	4.1%	12.0%	2.4%	
Divorced/separated	12.0%	21.1%	10.1%	
Widowed	43.6%	38.9%	44.7%	
Education				<.001
Less than high school	23.7%	34.4%	21.3%	
High school or equivalent	37.4%	34.2%	38.1%	
Some college	22.3%	24.2%	21.9%	
College or greater	16.6%	7.1%	18.7%	
Total net worth (in U.S. dollars)				<.001
Negative or zero	13.7%	28.9%	10.3%	
$1–49.9k	22.9%	33.1%	20.7%	
$50k–99.9k	9.9%	10.9%	9.7%	
$100k–249.9k	20.2%	17.1%	20.9%	
$250k–499.9k	13.1%	4.9%	14.8%	
$500k+	20.3%	5.1%	23.6%	
Proxy relationship				<.001
Spouse	26.5%	21.6%	27.6%	
Child/child-in-law	58.6%	53.3%	59.8%	
Other relative	11.9%	20.9%	10.0%	
Other/professional	2.9%	4.2%	2.6%	
Hospital death (ref = Home/facility)	28.5%	38.5%	26.3%	<.001
Death was unexpected (ref = Expected)	42.9%	56.6%	39.9%	<.001
Exit interview 2+ years after death (ref = 0–1)	34.2%	34.9%	34.1%	.789
Received palliative care	23.8%	22.0%	24.2%	.789

*Note.* Proportions are presented for categorical measures, and means (standard deviations) are presented for continuous measures. Chi-square (categorical measures) and *t*-tests (continuous measures) were conducted to evaluate statistically significant differences between White and Black decedents.

Statistically significant race differences denoted as:

* *p <* .05,

** *p <* .01,

*** *p <* .001.

### Perceived death quality

Table 2 shows the results for the OLS regression models predicting perceived death quality. The baseline (unadjusted) model shows Black–White differences only, with subsequent models incorporating measures of hospice usage (Model 2). The fully controlled model (Model 3) is expanded to include a two-way interaction between race and death quality (Model 4).

**Table 2.** Ordinary least squares regression models predicting death quality using health and retirement study exit survey data 2018, 2020, and 2022 (*N* = 2,498).

Variable	Model 1	Model 2	Model 3	Model 4
Black (ref = White)	1.00 (0.18)***	1.01 (0.18)***	1.19 (0.20)***	0.89 (0.23)***
Received hospice (ref = No)		0.01 (0.15)	−0.11 (0.17)	−0.27 (0.18)
Black × hospice				1.09 (0.41)**
Woman (ref = Man)			−0.15 (0.15)	−0.14 (0.15)
Hispanic (ref = No)			0.62 (0.29)*	0.61 (0.29)*
Age			0.03 (0.01)***	0.03 (0.01)***
Death during COVID-19 pandemic (ref = No)			0.05 (0.14)	0.06 (0.14)
Had no living will (ref = Had living will)			0.03 (0.15)	0.03 (0.15)
Children (ref = No)			0.05 (0.35)	0.06 (0.35)
Marital status (ref = Married/partnered)				
Never married			0.63 (0.43)	0.65 (0.43)
Divorced/separated			0.05 (0.27)	0.06 (0.27)
Widowed			0.35 (0.21)	0.36 (0.21)
Education (ref = Less than high school)				
High school or equivalent			0.09 (0.19)	0.09 (0.19)
Some college			0.35 (0.21)	0.36 (0.21)
College or more			−0.05 (0.24)	−0.04 (0.24)
Total net worth (in U.S. dollars)				
1–49.9k			−0.00 (0.24)	−0.01 (0.24)
50k–99.9k			−0.01 (0.30)	−0.00 (0.30)
100k–249.9k			0.08 (0.26)	0.09 (0.26)
250k–499.9k			−0.22 (0.29)	−0.22 (0.29)
500k+			0.01 (0.27)	−0.01 (0.27)
Proxy relationship (ref = Spouse)				
Child/child-in-law			−0.82 (0.22)***	−0.82 (0.22)***
Other relative			−0.25 (0.30)	−0.26 (0.30)
Other/paid/professional/don’t know			−0.39 (0.47)	−0.38 (0.47)
Hospital death (ref = Home/another facility)			−0.95 (0.17)***	−0.96 (0.17)***
Death was unexpected (ref = Expected)			0.38 (0.16)*	0.39 (0.16)*
Exit interview 2+ years after death (ref = 0–1)			0.21 (0.15)	0.20 (0.15)
Received palliative care (ref = Did not receive)			−0.54 (0.17)**	−0.54 (0.17)**
Constant	5.13 (0.08)***	5.13 (0.10)***	2.78 (0.82)***	2.83 (0.82)***
Adjusted R2	0.012	0.011	0.039	0.041

*Note.* Standard errors in parentheses.

* *p* < .05.

** *p* < .01.

*** *p* < .001.

**Figure 1.**
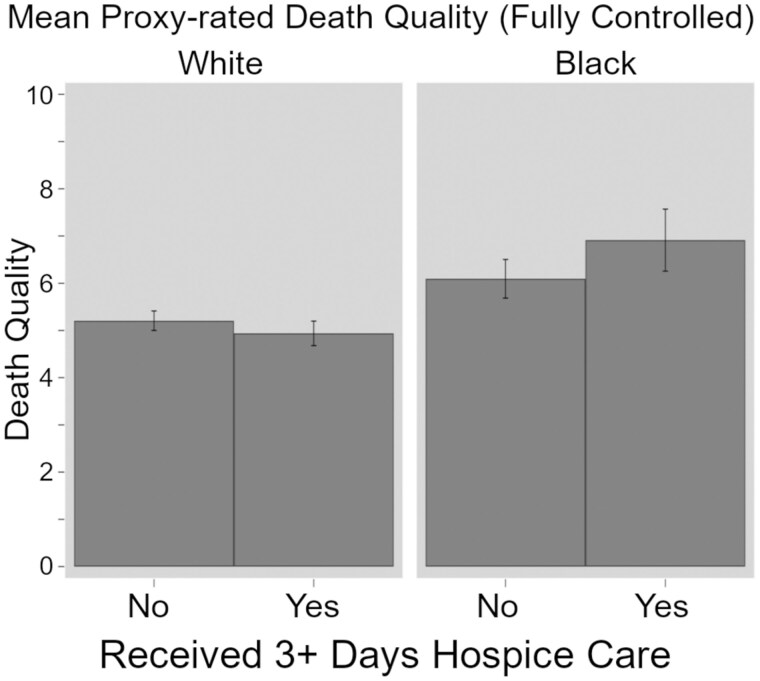
Mean proxy reported death quality measure by race and by decedents receiving or not receiving hospice care (fully adjusted model).

Across all models, Black decedents had overall better death quality based on their proxies’ perceptions. Interestingly, this advantage increases in magnitude with the addition of all covariates, with coefficients increasing by about 20% (from b = 1.0 to 1.19). The association between hospice use and perceived quality of death does differ significantly for Black and White decedents. Model 4 shows that among persons who did not use hospice services, Black decedents had death quality scores 0.89 points higher than their White counterparts. (*p* < .001). Conversely, among persons who did have hospice care at the end of life, this difference is estimated to be 1.09 points greater (*p* < .01). This suggests that hospice care was associated with improved perceived death quality for Black decedents but not White decedents. For ease of interpretation, fully adjusted quality of death scores for White and Black decedents on the basis of hospice use are presented in Figure 1.

Several control variables were statistically significantly associated with perceived death quality in the fully controlled model. Hispanic decedents have death quality scores that are 0.58 points higher than non-Hispanic decedents (*p* < .05), and for each additional year of age, there is a slight increase in death quality (a 0.03-point increase per year, *p* < .001). Deaths that were unexpected were associated with greater death quality, perhaps because decedents had a shorter duration of distressing symptoms (b = 0.39, *p* < .05).

Proxy respondents who are the decedent’s child rated death quality as significantly poorer (b = −0.86, *p* < .001), relative to spouse proxies. Hospital deaths were also associated with lower death quality, and, in our fully controlled model, a hospital death was associated with a 0.95-point lower death quality (*p* < .001). In contrast with receiving hospice care, palliative care services were associated with a 0.53-point lower death quality (*p* < .01). Deaths that occur during the COVID-19 pandemic are not associated with death quality relative to pre-COVID deaths.

### Perceived care concordance

Table 3 presents logistic regression models predicting whether decedents’ health care wishes were sometimes/usually met, according to their proxies. In the uncontrolled model, when compared to White decedents, Black decedents were significantly less likely to have their wishes always or usually met; the unadjusted odds (OR = 0.51, *p* < .001). In the fully controlled model (Model 3), this gap remained (OR = 0.74, *p* < .05). However, we find no evidence that the race disparity in care concordance differs on the basis of hospice use.

**Table 3.** Odds ratios from logistic regression predicting wishes being met using health, retirement study exit survey data 2018, 2020, and 2022 (*N* = 2,498).

Variable	Model 1	Model 2	Model 3	Model 4
Black (ref = White)	0.51 [0.40, 0.64]***	0.53 [0.42, 0.68]***	0.74 [0.56, 0.97]*	0.76 [0.55, 1.04]
Received hospice (ref = No)		1.36 [1.09, 1.70]**	1.08 [0.83, 1.40]	1.10 [0.83, 1.47]
Black × hospice				0.89 [0.51, 1.57]
Woman (ref = Man)			0.95 [0.76, 1.20]	0.95 [0.75, 1.20]
Hispanic (ref = No)			0.62 [0.42, 0.89]*	0.62 [0.42, 0.90]*
Age			1.01 [1.00, 1.02]	1.01 [1.00, 1.02]
Death during COVID-19 Pandemic (ref = No)			1.06 [0.85, 1.31]	1.06 [0.85, 1.31]
Had no living will (ref = Had living will)			0.58 [0.46, 0.74]***	0.58 [0.46, 0.74]***
Children (ref = No)			0.87 [0.52, 1.48]	0.87 [0.52, 1.48]
Marital status (ref = Married/partnered)				
Never married			1.15 [0.62, 2.10]	1.14 [0.62, 2.10]
Divorced/separated			0.98 [0.67, 1.43]	0.98 [0.67, 1.43]
Widowed			1.33 [0.97, 1.84]	1.33 [0.97, 1.83]
Education (ref = Less than high school)				
High school or equivalent			1.34 [1.02, 1.76]*	1.34 [1.02, 1.76]*
Some college			1.48 [1.07, 2.04]*	1.48 [1.07, 2.03]*
College or more			1.29 [0.89, 1.86]	1.28 [0.89, 1.86]
Total net worth (in U.S. dollars)				
1–49.9k			1.10 [0.78, 1.53]	1.10 [0.79, 1.53]
50k–99.9k			1.21 [0.79, 1.85]	1.21 [0.79, 1.85]
100k–249.9k			1.35 [0.93, 1.97]	1.35 [0.93, 1.97]
250k–499.9k			1.15 [0.75, 1.75]	1.15 [0.75, 1.75]
500k+			1.57 [1.03, 2.39]*	1.57 [1.03, 2.40]*
Proxy relationship (ref = Spouse)				
Child/child in law			1.08 [0.79, 1.50]	1.08 [0.79, 1.50]
Other relative			0.97 [0.62, 1.51]	0.97 [0.62, 1.51]
Other/paid/professional/don’t know			0.80 [0.41, 1.57]	0.80 [0.41, 1.57]
Hospital death (ref = Home/another facility)			1.01 [0.78, 1.30]	1.01 [0.78, 1.30]
Death was unexpected (ref = Expected)			0.73 [0.57, 0.92]**	0.73 [0.57, 0.92]**
Exit interview 2+ years after death (ref = 0–1)			1.29 [1.03, 1.63]*	1.29 [1.03, 1.63]*
Received palliative care (ref = Did not receive)			0.98 [0.76, 1.27]	0.98 [0.76, 1.27]
Constant	5.32 [4.73, 5.99]***	4.72 [4.08, 5.44]***	2.28 [0.69, 7.52]	2.27 [0.69, 7.48]
Adjusted *R* ^2^	0.013	0.016	0.058	0.058

*Note.* Standard errors in parentheses.

* *p* < .05.

** *p* < .01.

*** *p* < .001.

In Model 2, compared to those receiving no hospice care, receiving hospice care is associated with higher odds of having wishes met (OR = 1.36, *p* < .01), however, after including full controls, the association is no longer significant. Additionally, in the fully controlled models, we find that two core dimensions of a “good death”—having no living will compared to having a living will (OR = 0.58, *p* < .001) and dying unexpectedly compared to those who did not die unexpectedly (OR = 0.73, *p* < .01)—are associated with lower odds of care concordant with wishes. Additionally, decedents with a high school diploma or some college education were significantly more likely than people who did not receive a high school diploma to have their health care wishes always or usually met (OR = 1.34 and 1.48, respectively, both *p* < .05).

## Discussion

This study is the first, to our knowledge, to use the perceived death quality measure asked in the HRS Exit Survey from proxy respondents starting in 2018. These data, which enable us to explore prospectively the impact of decedent characteristics on proxy assessments of their end-of-life care, provide novel insights into racial differences in end-of-life experiences and expectations. Specifically, we investigated Black–White differences in two end-of-life measures (perceived death quality and perceived care concordance), taking into consideration hospice use. We found that Black proxies were less likely than White proxies to say the decedents’ care preferences were met at the end of life. This finding is consistent with prior research on Black–White differences in quality of care received over the life course. Our descriptive analyses further showed that Black decedents experienced other end-of-life disadvantages, including lower rates of hospice use and advance care planning, and higher rates of unexpected deaths and hospital deaths.

However, we also found that Black proxies offered more positive appraisals of the decedent’s quality of death, relative to White proxies, even after adjusting for socioeconomic, health, and family characteristics. This paradoxical finding complicates established narratives that Black Americans uniformly experience worse death quality than Whites. We believe our findings may reflect different cultural perceptions of what constitutes a “good death.” Black proxies’ high subjective appraisals of death quality relative to White proxies may reflect differences in expectations for the quality of care received at the end of life (Cain & McCleskey, 2019; Hauschildt, 2024). White middle-class persons may have particularly lofty or idealized expectations for their end-of-life experiences; as such, their proxies may offer negative appraisals of the decedent’s death quality, should it fall short of their high expectations or rigid definitions for what constitutes a “good death.” In contrast, Black proxies may have lower or more realistic expectations, a function of poorer quality health care experiences over the life course. An extensive literature documents that Black patients experience institutional racism in health care systems, discriminatory treatment in clinical settings, access to poorer quality health care facilities, and the receipt of care discrepant with their preferences (e.g., Aaron et al., 2021; Macias-Konstantopoulos et al., 2023; Williams et al., 2019; Fiscella & Sanders, 2016). As such, their generally positive appraisals of death quality may reflect these relatively low expectations for their care, or favorable comparisons of this death relative to other deaths that they witnessed, aligning with theoretical and empirical work suggesting satisfaction may be shaped by making comparisons to other externalized standards (Diener et al., 1985).

Additionally, our moderation analyses also revealed that one contextual factor—hospice use—moderated the observed association between race and perceived quality of care. Hospice use was associated with significantly better death quality assessments among Black decedents, but not among White decedents. This finding may similarly reflect Black–White differences in expectations, such that White persons—who have both higher rates of using hospice and more positive perceptions of hospice—may have unrealistically high expectations. In contrast, some studies suggest that Black persons hold very negative attitudes toward hospice, often based on misconceptions about hospice care (Rosenfeld et al., 2007; Tate et al., 2025). For Black individuals, exposure to hospice care may elicit more favorable appraisals due to the contrast with previously held negative expectations. Interestingly, we found no evidence that racial differences in end-of-life experiences changed in magnitude over the course of the pandemic.

Our results have potentially important implications for health care. Some health insurers are moving toward a “value-­based” payment model, in which practitioners’ reimbursement is based not only on clinical indicators of patient outcomes, but also subjective indicators, including patients’ satisfaction with care (Markowitz et al., 2022). The inclusion of patient experience metrics in value-based payment models is lauded by health care policy experts for its patient-centered approach. However, we caution that multiple indicators of patient experience should be included if indeed the subjective appraisals of Black proxies or patients misalign with the actual quality of care received.

Our findings also confirm that hospice care is associated with better subjective appraisals of quality of end-of-life care, especially for the proxies of Black decedents. These findings suggest that efforts to increase the use of hospice among Black older adults may be helpful for addressing systemic inequalities in health care. However, the beneficial association between hospice care and the quality of death may be underestimated, given some evidence suggesting that Black patients may receive lower quality hospice services than their White counterparts (Johnson, 2013; Welch et al., 2005). Additionally, significant labor shortages in the hospice and palliative care workforce during the COVID-19 pandemic (Kates et al., 2021) may have limited its usefulness during these survey waves, as recent qualitative research suggests that these labor shortages were most acute in lower-income neighborhoods and health care institutions. As such, the exacerbation of already existing disparities (David et al., 2025) and shortages in qualified hospice workers may have dampened the potential impact of hospice care on the quality of death for Black decedents. This highlights the need to not only expand access to quality hospice services but also ensure the care delivered is equitable across all communities.

When addressing disparities in death quality through expanded access to hospice care services, it is essential that the care patients receive is in concordance with their end-of-life wishes. Future research should incorporate measures of both objective and subjective death quality to understand how individual preferences in end-of-life treatments may align with dominant, structurally determined views of death quality, leading to a broader understanding of disparities in end-of-life care. Objective measures of death quality, such as pain management or treatment, can offer insight into whether care meets established clinical standards and generally agreed-upon, established death quality norms. In contrast, subjective measures, such as proxy-­reported death quality or care concordance, capture how the death was experienced and interpreted by patients and families. Relying on only one type risks overlooking important aspects of the dying experience, particularly in populations whose preferences may not align with dominant clinical or cultural norms.

### Limitations

While this study explores novel ways to identify death quality disparities, it has several limitations. First, due to sample size limitations, we are unable to evaluate death quality disparities for other minoritized populations in the United States. Second, some of our findings may also be driven by the utilization of proxy reports, which are necessary because all respondents are deceased and cannot directly report on their experiences. However, proxy assessments are not without their critiques; researchers have found that proxies tend to be more accurate when assessing objective indicators like coordination of health care, and less accurate when assessing subjective dimensions such as pain (McPherson & Addington-Hall, 2003). We cannot ascertain which particular dimensions of care weighed most heavily in the proxies’ assessments, although prior research suggests that lack of pain and physical comfort are considered foundational aspects of a “good death” (Steinhauser et al., 2000).

Future studies should incorporate additional clinical measures of death quality, such as end-of-life treatment and pain management, to obtain a more multifaceted measure of death quality, which may show different patterns of racial disparities in subjective and objective measures of end-of-life experiences. Additionally, future studies should examine the role of advance care planning in shaping death quality outcomes, as documented disparities in its utilization may lead to changes in end-of-life experiences across racial groups. While we include advance care planning as a control, we are unable to analyze it as a focal variable due to sample size constraints. It is also important to note that Black respondents account for 18% of our total sample, but they account for 30% of those who were dropped. This could be a source of systematic bias, whereas White respondents are more likely to have more highly engaged proxies. While we were unable to assess systematic differences between proxy respondents in this study due to sample size limitations, future research should examine how proxy characteristics may shape reported end-of-life outcomes to better understand the reliability of these measures across racial and social groups. Given that death quality is inherently difficult to measure, proxy assessments can provide uniquely valuable data. Finally, we cannot ascertain the race of the proxy, although, given relatively low rates of racial intermarriage in the HRS cohort, we presume that the decedent and proxy share a similar racial/ethnic background (Kalmijn, 1993).

## Conclusion

Understanding differences in death quality helps to identify health disparities that persist through the final stages of life. Our findings complicate dominant narratives about racial inequality in end-of-life care by showing that Black proxies report higher perceived death quality than White proxies, despite greater exposure to structural disadvantages. However, this should not be interpreted as evidence of more equitable care, as we also found Black decedents report lower rates of perceived care concordance. These positive appraisals may reflect lower baseline expectations due to a lifetime of systemic exclusion and unmet needs in health care. In this context, a death perceived as a good death may still fall short of basic standards for high-quality end-of-life care. At the same time, we find that hospice use is associated with increased perceived death quality, particularly for Black decedents—suggesting that expanding equitable access to high-quality hospice services may be one promising pathway for intervention. Medicare reimbursement policies that support end-of-life discussions, advance care planning, and culturally responsive communication may help ensure that care is more aligned with patients’ values and wishes.

## Data Availability

The Health and Retirement Study (HRS) data are public available at: https://hrs.isr.umich.edu/data-products. This study was not preregistered.

## References

[gbaf137-B5194389] Aaron, S. P. , Gazaway, S. B., Harrell, E. R., Elk, R. Disparities and Racism Experienced Among Older African Americans Nearing End of Life. Current Geriatrics Reports, 10, 157–166. 10.1007/s13670-021-00366-6. PMC8685164 34956825

[gbaf137-B55135783] Anhang Price, R. , Tolpadi, A., Elliott, M. N., Wang, S. E., Gozansky, W. S., Nguyen, H. Q., Teno, J. M., Ye, F., Timmer, M. A., Bradley, M. A. Surveying Family Caregivers to Assess Quality of End-of-Life Care in Medicare Advantage. Journal of Palliative Medicine. 10.1089/jpm.2025.0062 40336464

[gbaf137-B1] Bazargan M. , Bazargan-Hejazi S. (2021). Disparities in palliative and hospice care and completion of advance care planning and directives among non-Hispanic Blacks: A scoping review of recent literature. American Journal of Hospice and Palliative Medicine, 38, 688–718. 10.1177/1049909120966585 33287561 PMC8083078

[gbaf137-B2] Bernstein S. F. , Sasson I. (2023). Black and white differences in subjective survival expectations: An evaluation of competing mechanisms. SSM-Population Health, 21, 101339. 10.1016/j.ssmph.2023.101339 36785548 PMC9918793

[gbaf137-B3] Boyce-Fappiano D. , Liao K., Miller C., Peterson S. K., Elting L., Guadagnolo B. A. (2021). Preferences for more aggressive end-of-life pharmacologic care among racial minorities in a large population-based cohort of cancer patients. Journal of Pain and Symptom Management, 62, 482–491. 10.1016/j.jpainsymman.2021.02.001 33556498 PMC8339155

[gbaf137-B4] Braun U. K. , McCullough L. B., Beyth R. J., Wray N. P., Kunik M. E., Morgan R. O. (2008). Racial and ethnic differences in the treatment of seriously ill patients: A comparison of African-American, Caucasian and Hispanic veterans. Journal of the National Medical Association, 100, 1041–1051. 10.1016/S0027-9684(15)31442-5 18807433

[gbaf137-B5] Bugliari D. , Carroll J., Hayden O., Hayes J., Hurd M., Lee S., Main R., McCullough C., Meijer E., Pantoja P. (2023). *RAND HRS longitudinal file 2020 (V1) documentation. RAND center for the study of aging*. https://www.rand.org/content/dam/rand/www/external/labor/aging/dataprod/randhrs1992_2022v1.pdf. Date accessed October 14, 2024.

[gbaf137-B6] Cain C. L. , McCleskey S. (2019). Expanded definitions of the “good death”? Race, ethnicity and medical aid in dying. Sociology of Health & Illness, 41, 1175–1191. 10.1111/1467-9566.12903 30950077 PMC6786270

[gbaf137-B7] Carr D. (2012). Death and dying in the contemporary United States: What are the psychological implications of anticipated death? Social and Personality Psychology Compass, 6, 184–195. 10.1111/j.1751-9004.2011.00416.x

[gbaf137-B8] Carr D. , Luth E. A. (2019). Well-being at the end of life. Annual Review of Sociology, 45, 515-534. 10.1146/annurev-soc-073018-022524 PMC7731514 33311838

[gbaf137-B9] CDC COVID-19 Response Team. (2020). Geographic differences in COVID-19 cases, deaths, and incidence—United States, February 12–April 7, 2020. Morbidity and Mortality Weekly Report, 69, 465–471. 10.15585/mmwr.mm6915e4 32298250 PMC7755058

[gbaf137-B5838819] Colen, C. G., , Li, Q., Reczek, C., Williams, D. R. The Intergenerational Transmission of Discrimination: Children’s Experiences of Unfair Treatment and Their Mothers’ Health at Midlife. Journal of Health and Social Behavior, 60, 474–492. 10.1177/0022146519887347. PMC7810357 31912765

[gbaf137-B10] Conway S. (2012). Death, working-class culture and social distinction. Health Sociology Review, 21, 441–449. 10.5172/hesr.2012.21.4.441

[gbaf137-B11] David D. , Moreines L. T., Boafo J., Kim P., Franzosa E., Schulman-Green D., Brody A. A., Aldridge M. D. (2025). “Who you are and where you live matters”: Hospice care in New York city during COVID-19 perspectives on hospice and social determinants: A rapid qualitative analysis. Journal of Palliative Medicine, 28, 59–68. 10.1089/jpm.2024.0124 39451053 PMC12408888

[gbaf137-B12] Diener E. , Emmons R. A., Larsen R. J., Griffin S. (1985). The satisfaction with life scale. Journal of Personality Assessment, 49, 71–75. 10.1207/s15327752jpa4901_13 16367493

[gbaf137-B13] Emanuel E. J. , Emanuel L. L. (1998). The promise of a good death. The Lancet, 351, SII21–SII29. 10.1016/S0140-6736(98)90329-4 9606363

[gbaf137-B101] Fiscella K. , Sanders M. R. (2016). Racial and Ethnic Disparities in the Quality of Health Care. Annual Review of Public Health, 37, 375–394. 10.1146/annurev-publhealth-032315-021439 26789384

[gbaf137-B14] Hauschildt K. E. (2024). Whose good death? Valuation and standardization as mechanisms of inequality in hospitals. Journal of Health and Social Behavior, 65, 221–236. 10.1177/00221465221143088 36523154 PMC10267289

[gbaf137-B6838320] Health and Retirement Study, public use dataset. (2020). Produced and distributed by the University of Michigan with funding from the National Institute on Aging (grant number NIA U01AG009740). Ann Arbor, MI.

[gbaf137-B46371] Health and Retirement Study, public use dataset. (2022). Produced and distributed by the University of Michigan with funding from the National Institute on Aging (grant number NIA U01AG009740). Ann Arbor, MI.

[gbaf137-B15] Howarth G. (2007). Whatever happened to social class? An examination of the neglect of working class cultures in the sociology of death. Health Sociology Review, 16, 425–435. 10.5172/hesr.2007.16.5.425

[gbaf137-B16] Institute of Medicine. (2015). Dying in America: Improving quality and honoring individual preferences near the end of life. The National Academies Press. 25927121

[gbaf137-B17] Johnson K. (2013). Racial and ethnic disparities in palliative care. Journal of Palliative Medicine, 16, 1329–1334. 10.1089/jpm.2013.9468 24073685 PMC3822363

[gbaf137-B18] Kalmijn M. (1993). Trends in black/white intermarriage. Social Forces, 72, 119–146. 10.1093/sf/72.1.119

[gbaf137-B19] Kates J. , Gerolamo A., Pogorzelska‐Maziarz M. (2021). The impact of COVID‐19 on the hospice and palliative care workforce. Public Health Nursing, 38, 459–463. 10.1111/phn.12827 33111348

[gbaf137-B7048503] Kleinpell, R. , Vasilevskis, E. E., Fogg, L., Ely, E. W. Exploring the association of hospice care on patient experience and outcomes of care. BMJ Supportive & Palliative Care, 9, e13. 10.1136/bmjspcare-2015-001001. PMC5313381 27531840

[gbaf137-B20] Kris A. E. , Cherlin E. J., Prigerson H., Carlson M. D., Johnson-Hurzeler R., Kasl S. V., Bradley E. H. (2006). Length of hospice enrollment and subsequent depression in family caregivers: 13-month follow-up study. The American Journal of Geriatric Psychiatry, 14, 264–269. 10.1097/01.JGP.0000194642.86116.ce 16505131

[gbaf137-B21] Lo C. C. , Howell R. J., Cheng T. C. (2013). Disparities in Whites’ versus Blacks’ self-rated health: Social status, health-care services, and health behaviors. Journal of Community Health, 38, 727–733. 10.1007/s10900-013-9671-3 23483358

[gbaf137-B6538502] Macias-Konstantopoulos, W. L. , Collins, K. A., Diaz, R., Duber, H. C., Edwards, C. D., Hsu, A. P., Ranney, M. L., Riviello, R. J., Wettstein, Z. S. Sachs, C. J. Race, Healthcare, and Health Disparities: A Critical Review and Recommendations for Advancing Health Equity. The Western Journal of Emergency Medicine, 24, 906–918. 10.5811/westjem.58408. 37788031 PMC10527840

[gbaf137-B22] Markowitz W. , Kausar K., Coffield E. (2022). Relationship between patient experience scores and health insurance. Healthcare, 10, 2128. 10.3390/healthcare10112128 36360469 PMC9690600

[gbaf137-B23] McPherson C. J. , Addington-Hall J. M. (2003). Judging the quality of care at the end of life: Can proxies provide reliable information? Social Science and Medicine, 56, 95–109. 10.1016/s0277-9536(02)00011-4 12435554

[gbaf137-B24] Mirowsky J. (1999). Subjective life expectancy in the US: Correspondence to actuarial estimates by age, sex and race. Social Science & Medicine, 49, 967–979. 10.1016/s0277-9536(99)00193-8 10468401

[gbaf137-B25] National Institute on Aging. (2021). *What are palliative care and hospice care? *https://www.nia.nih.gov/health/hospice-and-palliative-care/what-are-palliative-care-and-hospice-care. Date accessed October 14, 2024.

[gbaf137-B26] O’Mahony S. , Kittelson S., Barker P. C., Delgado Guay M. O., Yao Y., Handzo G. F., Chochinov H. M., Fitchett G., Emanuel L. L., Wilkie D. J. (2021). Association of race with end-of-life treatment preferences in older adults with cancer receiving outpatient palliative care. Journal of Palliative Medicine, 24, 1174–1182. 10.1089/jpm.2020.0542 33760658 PMC8309420

[gbaf137-B2896121] Phelan J. C., & , Link, B. G. Is Racism a Fundamental Cause of Inequalities in Health? Annual Review of Sociology, 41, 311–330. 10.1146/annurev-soc-073014-112305.

[gbaf137-B8889706] Rosenfeld, P. , Dennis, J., Hanen, S., Henriquez, E., Schwartz, T. M., Correoso, L., Murtaugh, C. M., Fleishman, A. Are there racial differences in attitudes toward hospice care? A study of hospice-eligible patients at the Visiting Nurse Service of New York. The American Journal of Hospice & Palliative Care, 24, 408–16. 10.1177/1049909107302303. 17601837

[gbaf137-B27] Rosenfeld P. , Dennis J., Hanen S., Henriquez E., Schwartz T. M., Correoso L., Murtaugh C. M., Fleishman A. (2007). Are there racial differences in attitudes toward hospice care? A study of hospice-eligible patients at the Visiting Nurse Service of New York. American Journal of Hospice and Palliative Medicine, 24, 408–416. 10.1177/1049909107302303 17601837

[gbaf137-B28] Sayag M. , Kavé G. (2022). The effects of social comparisons on subjective age and self-rated health. Ageing & Society, 42, 2140–2153. 10.1017/S0144686X20002056

[gbaf137-B29] Sonnega A. , Faul J. D., Ofstedal M. B., Langa K. M., Phillips J. W., Weir D. R. (2014). Cohort profile: The health and retirement study (HRS). International Journal of Epidemiology, 43, 576–585. 10.1093/ije/dyu067 24671021 PMC3997380

[gbaf137-B30] StataCorp. (2023). Stata Statistical Software: Release 18. StataCorp LLC.

[gbaf137-B31] Steinhauser K. E. , Christakis N. A., Clipp E. C., McNeilly M., McIntyre L., Tulsky J. A. (2000). Factors considered important at the end of life by patients, family, physicians, and other care providers. JAMA, 284, 2476–2482. 10.1001/jama.284.19.2476 11074777

[gbaf137-B78602271] Tate, C. E. , Perez-Jolles, M., Scherer, L. D., Shiferaw, T., Mami, G., Matlock, D. D., Huebschmann, A. G. “Hospice was Created by the KKK”-Black Americans’ Perspectives on Hospice Care. Journal of Racial and Ethnic Health Disparities. 10.1007/s40615-025-02340-w. 40014286

[gbaf137-B33] Welch L. C. , Teno J. M., Mor V. (2005). End-of-life care in black and white: Race matters for medical care of dying patients and their families. Journal of the American Geriatrics Society, 53, 1145–1153. 10.1111/j.1532-5415.2005.53357.x 16108932

[gbaf137-B12591974] Williams, D. R., , Lawrence, J. A., Davis, B. A. Racism and Health: Evidence and Needed Research. Annual Review of Public Health, 40, 105–125. 10.1146/annurev-publhealth-040218-043750. PMC6532402 30601726

